# Evaluation of Three Different Laboratory Methods for Identification of *Pneumocystis jirovecii* Pneumonia (PCP) among HIV Positive Asymptomatic Prisoners

**Published:** 2019

**Authors:** Shohreh AZIMI, Azar SABOKBAR, Amir BAIRAMI, Mohammad Javad GHARAVI

**Affiliations:** 1. Department of Microbiology, Karaj Branch, Islamic Azad University, Karaj, Iran; 2. Department of Medical Parasitology and Mycology, School of Medicine, Alborz University of Medical Sciences, Karaj, Iran; 3. Department of Parasitology, School of Allied Medicine, Iran University of Medical Sciences, Tehran, Iran

**Keywords:** *Pneumocystis jirovecii*, Pneumonia, β-D-glucan assay

## Abstract

**Background:**

*Pneumocystis jirovecii* pneumonia (PCP) remains a leading cause of mortality among HIV-infected patients. The aim of study was to find out *P. jirovecii* in versatile group of HIV-positive patients prisoners.

**Methods:**

Overall, 102 HIV positive patients from Ghezel Hesar Prison, Karaj, Iran from October 2016 to March 2017 without any respiratory symptoms were selected with different medication histories against HIV and PCP. Microscopic and molecular (qualitative real-time PCR) examination were applied on sputum specimens and serological investigation (β-D-glucan assay for fungal diseases) carried out on patient’s sera.

**Results:**

Only 3 and 1 patients were positive for PCP by microscopic and molecular testing, respectively. Twenty-four (23.5%) and 78 (76.5%) out of 102 patients were seropositive and seronegative for fungi disease, respectively. Seropositive patients were older than seronegative subjects (*P*<0.001). Most of seropositive individuals showed less mean value of CD4 counts compared to seronegative group (*P*<0.001). Of 54 patients who were under HIV therapy, 13 were seropositive compared to 11 out of 24 seropositives who were no adhere to treatment (*P*<0.001). In terms of prophylactic antibiotic therapy against PCP, of 24 patients who received prophylaxis, 3 (12.5%) and 21 (87.5%) were seropositive and seronegative, respectively (*P*<0.001). On the contrary, among 78 patients who did not receive prophylaxis, 21 (27%) and 57 (73%) belonged to seropositive and seronegative patients, respectively (*P*<0.001).

**Conclusion:**

There was no strong evidence for PCP infection/disease among symptomless, HIV positive patients. According to their mean CD4 counts, the hypothesis for being negative in a majority of applied tests would be the absence of severe immunosuppression in the patients.

## Introduction

*Pneumocystis jirovecii* belongs to *Pneumocystidaceae* family, formerly known as *P. carinii*, which is an opportunistic fungus causing severe *Pneumocystis* pneumonia in immunocompromised subjects, especially in of HIV/AIDS patients. Despite prophylaxis against *P. jirovecii* in high-risk patients, it continues to be an agent for opportunistic pneumonia. PCP remains the leading cause of mortality among HIV-infected subjects for those who are not under antiretroviral therapy. About 20% of AIDS patients have been infected with PCP and the mortality rates range from 10% to 40% in those with life-threating pneumonia followed by respiratory failure quickly in a few days (1, 2) The clinical manifestation is non-specific and radiological findings are unspecific, as they may overlap with other lower respiratory tract infections, therefore, they do not have sufficient diagnostic sensitivity and specificity to be helpful (3). Thus, an ultimate identification requires laboratory diagnosis of the organism in respiratory samples (4).

Nowadays, biological diagnosis of PCP in the laboratory is mandatory, which depends on the microscopic observation of respiratory specimens of trophic forms or cysts after using appropriate staining techniques. Staining of bronchoalveolar lavage remains the most commonly used technique. However, microscopic identification is troublesome and needs specific expertise and due to the absence of sensibility for low fungal load, therefore, it could be falsely negative when noninvasive pulmonary specimens are used (5).

Moreover, several antibody-based staining methods are existed based on direct IF antibody stains with *P. jirovecii* monoclonal antibodies, which demonstrate higher sensitivity and specificity in comparison with the traditional staining techniques. Serum measurement of (1–>3)-β-D-glucan (BG) is based on the level of this polysaccharide which is present within the cell wall of pneumocystis and other fungi (6). Measurement of blood levels of BG has emerged as a noninvasive way and potentially useful diagnostic method in immunocompromised patients, including those with HIV (7, 8) and several tests commercially available for the detection of BG.

The use of PCR in the diagnosis of *Pneumocystis* infection has enhanced the laboratory identification because of its high sensitivity and specificity (9). Quantitative real-time PCR (qPCR) has frequently used to rule out the PCP diagnosis due to its high negative predictive value (NPV) which is approximately 100% (5). Diverse polymerase chain reaction (PCR) methods applying arrays of gene targets in the *Pneumocystis* genome have been aimed as potential diagnostic markers. Molecular methods applying PCR techniques are more sensitive than staining procedures for the detection of *P. jirovecii* in respiratory specimens (10). Real time PCR allows precise quantification of DNA and the potential for distinguishing between carriers of *P. jirovecii* and clinical illness based on fungal load with high sensitivity and specificity in comparison with gold standard staining methods (1, 11). Recently, several companies have produced commercial kits, however, only some of studies evaluated their efficiencies and none compared them (12).

The aims of the present study were to investigate the possible existence of PCP in HIV positive patients using three different approved laboratory approaches and to compare their application for the identification of PCP in different groups of asymptomatic HIV positive prisoners.

## Methods

### Patient’s samples

This cross-sectional study was conducted in Alborz University of Medical Sciences and Fardis Central Laboratory, Karaj, Iran in collaboration with Iranian Prisoners Health and Cure Organization from October 2016 to March 2017.

The study protocol was approved by Iranian Prisoners Health and Cure Organization Ethical Committee. All participants were HIV-1 positive prisoners. They had past histories of intravenous drug injection, sharing needles, high-risk sexual relationship and tattooing with different frequencies. However, the establishment of previous rout of transmission for each patient according to patient’s clinical file was problematic, as a majority showed some common source of transmission. According to Iranian Center for Disease Control (CDC) guidelines, all HIV-1 positive patients who fulfill the criteria for antiviral therapy receive tenofovir (TDF), lamivudine (LAM) and efavirenz (EFV). However, previous guideline was based on TDF, LAM and *zidovudine* (ZDV). Moreover, they receive *trimethoprim/sulfamethoxazole* (TMP/SMX) for the aim of prophylaxis against PCP if they show CD4< 200 cells/mm^3^. Random selection of 102 HIV-positive patients was carried out according to inclusion criteria including HIV positive patients regardless of being under TMP/SMX therapy with the absence of any signs and symptoms of any respiratory infection. These patients were categorized based on their antiretroviral therapy status (Table 1). One group were consisted of patients who had received TDF, LAM and ZDV (n= 21, 20%). Another group had received TDF, LAM and EFA (n=33, 32%). The last group composed of patients who did not receive medication at the time of investigation due to nonadherence to therapy before and after imprisonment (n= 48, 47%). This latter group had a mystifying clinical profile. Health authorities could not be able to find out the exact previous history of their medication. However, all had been received antiretroviral medication in the past. For all patients, the following data were additionally analyzed: demographics, laboratory values, medical history (including chronic lung disease) and antibiotic therapy. Other medical information was extracted from the medical files.

Overall, 102 expectorated sputum and serum samples were taken for *Pneumocystis* investigation. Sputum specimens were collected at the time of initial interview. After patients consent, questionnaires were completed by interviewer. Then, sputum specimens were collected by having the patient cough 5 times against a semi-closed mouth, spitting the specimen in a sterile specimen cup. The samples were shipped on dry ice to the Fardis Central Laboratory for molecular analysis.

### Microscopic Examination

All sputa were liquefied by addition of 4% NaOH solution, neutralized by sterilized distilled water after 3 min incubation. Slides were prepared according to the instructions of the manufacturer (Diff-Quick, Avicenna Research Institute, Tehran). Briefly, the smear was fixed for 75 sec with A solution. Then, slides were dipped in B solution for 60 sec, were rinsed with distilled water, and then were dipped 15 sec with C solution. After rinsing with distilled water, slides were examined under oil immersion.

### Serology

Two procedures could be used to measure the (1→3)-ß-D-glucan; the end-point assay and the kinetic rate assay. In this study, we used the end-point assay. Blood samples for pneumocystis serology were collected in endotoxin-free tubes to avoid endotoxin contamination and were tested using the (1→3)-ß-D-glucan assay (Glucatell, Cape Cod, East Falmouth, MA, USA). The assay was performed according to the protocol supplied by the manufacturer. 50μL of pachyman (Reagent Grade Water to the vial of glucan standard) at 40, 20, 10, and 5 pg/mL were used for the standard curve. 50μL of Reagent Grade Water was used as blank. Glucatell was reconstituted with one vial of Glucatell reagent using 2.8 mL of Pyrosol and subsequently, 50 μL of this mixture was added to each sample, standard and blank wells. Microplate was incubated at 37° C for 30 min. The reactions were stopped by adding of 50μL of sodium nitrite, 50μL of ammonium sulfamate together with 50 μL of N-(1-Napthyl) ethylenediamine dihydrochloride, respectively. Microplates were placed in the microplate reader and subsequently, their optical densities were read at 540–550 nm. The desired cut off for being positive in this assay was 80 pg/mL according to the levels proposed (13–20).

### Molecular Testing

Sputum specimens were treated before extraction. They were collected into 50 ml falcon tubes, homogenized after mixing equal volume of 4% NaOH solution. The contexts mixed intensely with a tube rotator for 5–20 min and then centrifuged at 3000 rpm for 15 min. The supernatants discarded and the pellets resuspended and centrifuged at 12000 rpm for 5–10 min. After discarding the supernatant, the pellets were collected into a 1.5ml tube for extraction.

For DNA extraction, 300 μL of lysis solution was added to each tube of samples, mixed and incubated for 5 min at 65 °C. Prec Sol (400 μL) was added, mixed and centrifuged at 13000r/min for 5 min. Two washing steps were carried out using 500μL and 200μL of Wash Sol, respectively. In each latter step, tube contents centrifuged at 13000r/min for 60 sec and supernatant was discarded. All tubes were incubated at 65 °C for 5 min. Finally, pellets were resuspended in 50μL of RE-buffer, incubated at 65 °C for 5 min, centrifuged at 13000 for 60 seconds, then, the supernatants were kept in −80 °C until amplification step.

Qualitative real-time PCR was performed on all extracted samples using commercial kits (Sacace, Via Scalabrini, Italy), according to the manufacturer’s recommendations. The assay contains an internal amplification control DNA sequence, which is not present in *Pneumocystis* and other fungal, bacterial or human genomes, to exclude the presence of any undesired amplicons at the end.

All PCR assays were performed with precautions against cross-contamination. To prevent carry-over contamination during PCR, each step of the procedure was performed in a separate room with dedicated equipment and directional flow from the beginning of the procedure to the end. Negative controls containing serum or water were also included in each extraction run, and an extra negative control containing water was included.

### Statistical analysis

Statistical analysis was performed by using the Statistical Package for Social Science (SPSS 21, SPSS Inc., Chicago, Illinois, USA). Data were expressed as percentages for categorical variables and means± standard deviations (SDs) for continuous variables. The intergroup differences of numerical values were performed by using the Student’s *t*-test. Categorical variables were expressed as percentages, and differences between groups were judged for significance using the chi-squared test. For all comparisons, *P*<0.05 was considered as statistically significant.

## Results

All patients were male with a mean age of 39±7 yr old. The mean CD4 counts of subjects were 449±296 cells/mm^3^. Totally, 51 (50%) of patients had coinfection, 46 (45%) were coinfected with viral hepatitis B or C and 42 (41%) were positive for anti-HCV, of whom 3 were triple-infected with *tuberculosis* (TB). Only one patient was HBV positive. Five patients were infected by TB. Among the patients, 54 (53%) were under antiviral therapy for HIV-1 (Table 1). 78 (76.5%) of patients did not receive prophylactic antibiotic therapy for PCP at all.

Only 3 (2.9%) subjects were positive for PCP in direct microscopic examination. No more than one patient was positive for PCP in molecular testing. The respiratory secretion of this positive patient was not positive for either direct microscopic observation or serology (results not shown). Due to the few numbers of patients who was positive in two latter assays, the statistical analysis and comparisons between negative and positive groups were irrational, therefore, only the details of serological analysis as follows:

Table 1 shows the results of serologic data for patients studied. Twenty-four (23.5%) and 78 (76.5%) out of 102 patients were seropositive and seronegative for fungi disease, respectively. The mean age of participants was 39±7 yr old. This value was 42±9 yr old and 38±7 yr old for seropositive and seronegative patients, respectively; showing a significant association between the age and being positive for fungi disease including PCP (*P*<0.001, Table 1). Seropositive and seronegative individuals showed 369±251 and 473±306 mean value of CD4 counts, respectively with a substantial association difference in between (*P*<0.001, Table 1). Three of seropositive patients were also positive in direct microscopic examination.

**Table 1:** Results of demographic, clinical and serological characteristics of patients studied

***Variable***	***All Patients n= 102(%)***	***Fungi Seropositive n= 24(%)***	***Fungi Seronegative n= 78(%)***	***P-Value***
Sex, n (%)				
Male	102	24 (100)	78 (100)	-
Age(yr)	39±7	42±9	38±7	<0.001
Coinfection, n (%)				
HBV	1 (0)	0 (0)	1 (1)	0.092
HCV	42 (41)	5 (20)	37 (47)	
TB	5 (4)	2 (8)	3 (3)	
HCV+TB	3 (2)	0 (0)	3 (3)	
No	51 (50)	17 (70)	34 (43)	
CD4 Count, mean±SD	449±296	369±251	473±306	<0.001
PCP prophylaxis use, n (%)				
Yes	24 (23.5)	3 (12)	21 (26)	<0.001
No	78 (76.5)	21 (87)	57 (73)	
Anti-viral drug use, n (%)				
LAM+TDF+EFA	21 (20)	3 (12)	18 (23)	<0.001
LAM+TDF+ZDV	33 (32)	10 (41)	23 (29)	
No Therapy	48 (47)	11 (45)	37 (47)	

Note: HBV, *Hepatitis B Virus*; HCV, *Hepatitis C Virus*; TB, *Tuberculosis*; LAM, lamivudine; TDF, tenofovir; EFV, efavirenz; ZDV, zidovudine

There was a strong correlation between both groups who either were on therapy as well as those who did not receive treatment and their PCP serostatus; 78 were seronegative versus 24 who were seronegative, respectively (*P* <0.001, Table 1).

Finally, 51 (50%) of patients were shown to be coinfected by either viral hepatitis (B and C) or TB. Only 7 (13.7%) cases belonged to seropositive group and the remaining 44 coinfected subjects were seronegative.

## Discussion

PCP is a life threating complication, which affects HIV/AIDS patients. PCP diagnosis in these high-risk patients’ remains challenging. Despite the existence of several laboratory methods, still, the differentiation between infection and colonization of PCP in asymptomatic patients or in those who receive prophylactic regimen is unknown. Therefore, the preliminary aim of this study was to investigate the existence of *Pneumocystis* in respiratory specimens of HIV-infected patients using a qualitative real-time PCR and secondly, to compare the valuableness of microscopic, serologic and molecular approaches in those patients. In the present study, only 4% (PCR and microscope) of HIV-positive patients showed evidence of PCP using PCR and microscopic tests.

One of the obstacles for successive antiretroviral therapy composed of in adherence of some patients to the medication. This is especially true for people who inject intravenous drugs. In this survey, 48 (47%) of studied group did not compliance with the therapy; a majority of whom were injecting drug users (results not shown). Interestingly, we did find a strong correlation between being seronegative for BG assay in the absence of treatment in this group (*P*<0.001). In addition, for those who were under therapy, the same was true for being seronegative. On the other hand, 13 and 11 of subjects were seropositive in those who received therapy versus those who did not, respectively (*P*<0.001). We appreciate that even those subjects who were in no therapy group were adhered to treatment in the initial period of their disease. Therefore, a majority of HIV patients were seronegative regardless of antiretroviral therapy.

We applied a sensitive molecular assay for the identification and qualification of *Pneumocystis* in these patients. However, only one case was positive for *Pneumocystis*. As this patient was not positive in both serologic and microscopic tests, he was an asymptomatic PCP carrier in the absence of any clinical features, assigned as being an asymptomatic *Pneumocystis* carrier. This hypothesis had already been proposed by others that rates of 2%–21% of HIV patients are positive for PCP by PCR (21, 22). Due to the low number of PCR-positive patients in this study, we could not be able to extend this finding and to draw a definite conclusion for other PCP positive patients. Twenty-four (23.5%) of patients were positive for fungi disease in BG assay. None showed clinical evidence for respiratory infections including PCP. Despite the fact that serum BG has outstanding discriminatory power for the diagnosis of PCP in patients with AIDS, an unavoidable limitation of this test is that it is not specific to *Pneumocystis jirovecii* (7). Moreover, a positive BG test should be confirmed with a *Pneumocystis*-specific assay (23) Nevertheless, in the present study, none of patients showed evidence of PCP clinical manifestation. Therefore, two possibilities exist. Either the patients were PCP asymptomatic carriers, or they had been infected (clinically or sub-clinically) by other fungal species other than PCP. Seropositive individuals had less mean CD4 counts than seronegative subjects did (369±251 and 473±306 cells/mm^3^ respectively, *P*<0.001). Altogether, the mean value of CD4 was above 200 cells/mm^3^ for all patients. Value of 200 cells/mm^3^ has been considered as a standard cut off for PCP chemoprophylaxis start point (24). Therefore, the hypothesis for being negative in a majority of applied tests would highlight the absence of severe immunosuppression of patients, signifying the correlation between this assay and the levels of immunosuppression and vulnerability to fungal/PCP infection (25).

A majority of individuals (78, 76.5%) did not have a history of receiving PCP chemoprophylaxis. Moreover, a majority of both chemoprophylaxis and no-chemoprophylaxis recipients were seronegative (*P*<0.001). This finding flourished a strong correlation between being negative for serology and the presence of this type of regimen, designating the importance of prophylaxis in these high-risk patients (26).

We could not find any correlation between the coinfection with hepatitis B, hepatitis C or TB) and noncoinfected HIV patients in terms of serological status for PCP (*P*-value= 0.092). This finding deserves further investigation, especially for those who coinfected by TB as a superimposed factor on a PCP basis (27–31).

This study had some limitations. Firstly, the sample size of HIV-positive patients was small. This prevents making a definite conclusion. Secondly, past medical history among the group who received TDF, LAM and EFA was ambiguous. Thirdly, the β-D-glucan assay was unable to differentiate and to specify PCP from other fungi.

## Conclusion

Using three different laboratory approaches, there was no strong finding for PCP infection/disease among symptomless, HIV positive patients. Despite majority of HIV patients did not receive chemoprophylaxis against PCP, they showed no sign of microscopic, serologic and molecular evidence of PCP infection due to the absence of severe immunosuppression. These patients should be followed up carefully due to the possibility of being infected subclinically with PCP.
